# Delayed Cerebral Ischemia After Aneurysmal Subarachnoid Hemorrhage: The Role of the Complement and Innate Immune System

**DOI:** 10.1007/s12975-024-01290-5

**Published:** 2024-08-21

**Authors:** Jose Javier Provencio, Sonya Inkelas, Mervyn D. I. Vergouwen

**Affiliations:** 1 Department of Neurology, University of Virginia, Charlottesville, VA USA; 2 Department of Neurology and Neurosurgery, UMC Utrecht Brain Center, University Medical Center Utrecht, Utrecht University, Utrecht, The Netherlands

**Keywords:** Subarchnoid hemorrhage, Innate inflammation, Neutrophils, Complement, Microglia

## Abstract

Specific inflammatory pathways are important in the development of delayed cerebral ischemia after aneurysmal subarachnoid hemorrhage. Understanding the specific pathways of inflammation may be critical for finding new treatments. Evidence is accumulating that innate inflammatory cells and proteins play a more important role than cells of the adaptive inflammatory system. In this work, we review the evidence from clinical and preclinical data regarding which cells of the immune system play a role in DCI with particular emphasis on the bone-marrow-derived cells monocytes and neutrophils and the brain parenchymal microglia. In addition, we will review the evidence that complement proteins, a non-cellular part of the innate immune system, play a role in the development of DCI.

## Introduction

Inflammation, like the nervous system, is a very complex system of unique cell types that work in lineage and context-dependent manners. Cells in the immune system have both constitutive properties (T cells always express the T cell receptor) and context-dependent behaviors (monocytes express adjacent cell-activating cytokines early in an insult and modulatory cytokines as repair starts). As an analogy, GABAergic inhibitory neurons (a constitutive subset of neurons which make up 10–15% of total neurons) have different roles in the hippocampus (memory formation) than the same lineage of neurons in the motor cortex (motor control). Therefore, the commonly used phrase “inflammation plays a role in subarachnoid hemorrhage” is as meaningful as the statement “neurons play a role in fine motor movements.”

Instead, specific, coordinated sets of inflammatory cells and proteins work together to achieve specific outcomes in different environments [1]. The same inflammatory cell in two different environments can act in different ways, expressing different cytokines and exhibiting different effector functions. This is most evident in the setting of infectious versus sterile insults. Although much of our understanding of the immune system is based on investigations of how inflammation works to clear infections, the role of the inflammatory system to mitigate injury and enact repair in non-infectious settings is less-well studied, and only relatively recently appreciated [2].

In the setting of delayed cerebral ischemia (DCI) after aneurysmal subarachnoid hemorrhage, there has been an understanding that “inflammation” plays a role in the development of DCI and the associated vasospasm since the 1970s [3–7]. What parts of the immune system and how those actor cells achieve the cognitive and executive dysfunction apparent in DCI is only recently being investigated.

In this review, we will investigate what is known about cells in the immune system that play a role in the development of DCI. For a comprehensive review of post-SAH inflammation, we refer to a previous review [8].

## Innate Versus Adaptive Immunity

To begin, it is important to understand how the constitutive components of the immune system are characterized. The immune system is composed of two parts: the innate immune system and the adaptive immune system, named by the component cells’ ability to adapt to pathogen-specific motifs. Innate immunity is the body’s non-specific initial defense against pathogens that is based on genetically determined recognition of danger or pathogen-associated molecular patterns (DAMPS and PAMPS, respectively). Through physical, chemical, and cellular mechanisms, it works to isolate pathogens (often infectious but sometimes injury) and prevent damage from spreading. In the broadest sense, innate immunity works through physical barriers such as the skin, chemical substances such as acids and enzymes in the gut, and activation of inflammatory cells such as macrophages, neutrophils, monocytes, natural killer cells, and mast cells.

Adaptive immunity traditionally comes after the innate response, particularly in infection. Aptly named, it targets specific pathogens and adapts to recognize them. This involves: 1) the presentation of processed antigens by professional and non-professional antigen-presenting cells such as dendritic cells and macrophages, 2) the development of specific T and B lymphocytes based on this presentation, and 3) the clonal expansion of those cells to attack pathogens. Adaptive immune cells typically last longer than those of the innate system and retain a subset of long-lived memory T cells that allow rapid clonal re-expansion when subsequently challenged by the same pathogen.

The model of innate immune cells non-specifically targeting pathogens or damage initially followed by more fine-tuned adaptive responses is proving to be more common for infectious agents. Work in autoimmunity as well as sterile injury shows prolonged effects of innate immune cells in the pathogenesis [9–11].

In addition to the cellular actors in the innate immune system, a number of proteins play an outsized role in that innate response. Chief among these is complement, a cascade of proteins that are triggered by a number of non-specific inflammatory signals [12]. By convention, the complement proteins are named with the letter C and numbers 1 through 9 (with small letter postscripts to denote split or modified proteins). Complement activation works through three pathways: the classical, alternative, and lectin  complement pathways (LCP). The classical pathway starts with the pathogen-binding C1q which is cleaved into C2b and C4b, that subsequently bind together to form the C3 convertase complex, the hinge complex that limits the rest of the reaction. The lectin pathway is initiated by mannose-binding lectin special protein (MBL). Conversely, the alternate pathway is not initiated through C1q but can start with any C-protein upstream of the C3 complex. Downstream, hydrolysis of C3 leads to the production of C3a and C5a (potent anaphylatoxin) and C3b which lead to the activation of other complement proteins. Of note, an important effector function of complement is that it forms a “late” event called the membrane attack complex (MAC). The MAC is a complex of C5b-C9 which forms a pore in the lipid bilayer membrane of damaged cells or bacteria that structurally destroys them by opening their cellular contents to the environment.

## Innate Immune System in the Brain

The brain is unique from other organs in the body in that traditional innate immune cells such as monocytes, macrophages, and neutrophils are not found in the central nervous system in health. The best categorized innate immune cell in the brain is the microglia which shares some traits with tissue macrophages but come from a distinct embryonic lineage [13]. In addition, astrocytes, the major structural cell of the brain, and oligodendrocyte precursor cells (OPCs) also exhibit aspects of immune responses [14, 15]. The glial cell response in brain injury has previously been reviewed [16]. Interestingly, during sterile injury in the absence of necrosis, some systemic (bone-marrow derived) innate immune cells, particularly neutrophils, do not infiltrate the brain parenchyma but remain in the meninges [17].

How the systemic innate inflammation central nervous system inflammatory system ‘cross-talk’ is an area of intense research [18]. Neurons, astrocytes, OPCs, and microglia express various Toll-like receptors (TLR) which sense cell surface receptors for DAMPs, complement proteins, and other immune proteins shared with the systemic system [19–23]. The glymphatic-to-lymphatic pathway in the CNS can take cytokine/chemokine/DAMP information to the cervical lymph nodes and is likely a major mechanism for systemic immune system activation in isolated brain injuries [24, 25].

On the effector side, the blood–brain (BBB) and blood-CSF barriers prevent the unimpeded entry of immune cells into the brain parenchyma, but injury/inflammation on either side of the barrier has been shown to disrupt the integrity of these systems [26, 27]. Cytokines, chemokines, and complement can cross the intact BBB and may signal brain inflammation. How systemic inflammation leads to downstream brain damage is not clear, but microglia and astrocytes have been implicated [28–30]. In addition, brain-directed systemic inflammation modulation can occur through the vagus nerve (via the acetylcholine alpha-7 nicotinic acetylcholine receptor) which can be dysregulated in brain injury leading to accentuated inflammatory responses [31, 32].

## Inflammation in SAH

All acute brain injuries involve inflammation; this is true for SAH as well. Acute inflammation after the rupture of an aneurysm can be caused by acute increased intracranial pressure (ICP), blood flow arrest at the time of high ICP and subsequent reperfusion, the effects of blood cell degradation or clotting factors in the cerebrospinal fluid (CSF), or acute hydrocephalus due to obstruction of the CSF outflow from the brain [33]. This early innate inflammation is part of what is now commonly termed “early brain injury (EBI),” a process that occurs in the first 72 h after bleeding [34, 35].

Four days to 2 weeks after SAH, approximately 30% of the patients have a delayed deterioration with focal neurological signs or a decrease in the level of consciousness which can lead to permanent disability, long-term cognitive deficits, and even death. Traditionally, this event has been referred to as cerebral vasospasm, but currently delayed cerebral ischemia is the accepted term [36]. Although the majority of SAH patients have angiographic vasospasm, neurological deterioration from delayed cerebral ischemia only occurs in a subset of patients with angiographic vasospasm. Conversely, delayed cerebral ischemia can occur in the absence of vasospasm. In addition, human trials of vasodilators such as endothelin-1 antagonists decreased the incidence of vasospasm but did not improve outcomes [37, 38]. EBI has been implicated in the pathway that leads to this delayed deterioration although the mechanism is still unclear. Inflammation, starting at the time of the aneurysm rupture, appears to be the likely precipitant of DCI [39]. The critical parts of the inflammatory response are becoming clearer.

Inflammatory changes in the blood and CSF of patients with SAH are associated with DCI [40]. Cytokine analyses and analysis of total lymphocytes does not improve our understanding of what parts of the inflammatory system are critical [41–44]. More recently, ratios of neutrophils or monocytes to lymphocytes have been studied in patients, but suffer from the assumption that an increased number of a particular cell type is important for pathology [45–47]. Interestingly, for years, systemic innate immune cells (of bone marrow origin) have been implicated in the pathology of DCI in animal models, but because of the prevailing understanding that innate immune reactions are early and time-limited, they have been largely discounted. CD11b, a surface marker found most densely on cells of the innate immune system such as neutrophils, monocytes, and natural killer cells, is a critical determinant of vasospasm in murine models of SAH [48–51]. In addition, sequestration and deactivation of monocytes in the blood of animal with SAH improved cerebral vasospasm [52].

Recently, elegant evaluation of cytokines, complement, and other immune proteins using large data set analysis has become a more valuable tool for understanding what cellular responses are important in DCI after SAH [53]. Although individual cytokines have many functions in both the innate and adaptive system, networks of cytokines tend to align with specific environments and effector responses. In patients with SAH, the association of a number of cytokines in blood including TNF⍺, IL-6, IL-17A, and IL1RA is more closely associated with the innate immune system than the adaptive [54].

Direct evidence of innate immune pathways leading to delayed cerebral vasospasm and late cognitive dysfunction come from animal studies in models of SAH. Studies have shown that monocytes and neutrophils play a role in the development of delayed cerebral vasospasm and delayed spatial memory deficits (DCV/DSMD). Inhibition of monocytes and neutrophils using the depleting Ly6G-C antibody prevent the onset of DCV/DSMD when given prior to the SAH, while the specific neutrophil depleting antibody (targeting Ly6G) does not work when given early but does prevent murine DCV/DSMD when administered 3 days post SAH [17, 55]. This suggests an early role for monocytes and a more prominent role of neutrophils 3 days after SAH. Infiltration of monocytes into the brain in a murine model of SAH occurs one day after the hemorrhage. Interestingly, a subset of infiltrating monocytes, Ly6C^hi^ monocytes improve outcome on behavior scoring, and functional dexterity suggesting the role of monocytes after SAH is complicated [56]. A major limitation of studying infiltrating monocytes in the setting of SAH is the difficulty discriminating them from microglia, pericytes and perivascular macrophages [39].

Neutrophil involvement in DCV/DSMD after SAH is based on studies that either deplete or genetically alter neutrophil function in mice. Specifically, neutrophil depletion 3 days after SAH in a model of mild SAH shows decreased DCV/DSMD [17]. Genetic depletion of myeloperoxidase, a neutrophil effector enzyme that acts as a signaling molecule and catalyzes the reaction of halides with superoxide to make bleach, prevents the development of DCV/DSMD [57]. MPO inhibitors have been tried in clinical trials of non-neurological diseases and may be potential therapeutic agents.

The complement system plays an important role in the brain’s inflammatory response. Signaling molecules C3a and C5a, in particular, are anaphylatoxins that mediate inflammation by inducing vasoconstriction and initiating phagocytosis by inflammatory cells both in the brain and from systemic inflammation [58]. C5a levels in CSF peak on day 1 after SAH and gradually decrease [59]. In animal studies, mice lacking C5a receptors and mice treated with C5 antibodies have reduced brain injury. However, a phase 2a clinical trial showed that eculizumab, a monoclonal antibody that inhibits the cleavage of C5 (and, sequentially, prevents the formation of C5a and the MAC), did not result in a statistically significant decrease of C5a and the MAC in CSF [60]. However, in serum, eculizumab decreased C5a concentration and functional complement activity of the classical, alternative, and lectin pathways of complement activation, while interleukin-6, interleukin-10, sC5b-9, and C-reactive protein concentration in serum did not differ between both groups. It remains to be investigated why the effect of eculizumab was discrepant in serum and CSF. Although eculizumab did not decrease the incidence of DCI or poor outcome, it needs mentioning that this small phase 2a trial was not powered to detect such differences.

In addition, increased levels of LCP initiators such as ficolin-1–3 and MBL in plasma have been shown to correlate with poor prognosis [61]. Plasma concentrations are elevated during the first week after SAH and decrease over the next two. Levels in the CSF were predictive of poor functional outcomes but not DCI.

In addition to the systemic inflammatory system, the brain’s inflammatory system has also been implicated in the pathology of DCI. The implication of microglial activation as a driver of brain injury after SAH is based on “microglial activation,” although microglial activity is difficult to evaluate [16, 62–65]. As stated above, a major limitation of this work is based on the difficulty discriminating microglia from infiltrating monocytes, pericytes, and perivascular macrophages. Some studies have noted an expansion of microglia after SAH and others have noted an infiltration of monocytes using the same cell surface markers to identify both. Single-cell RNA sequencing technology holds hope for distinguishing these two cell types, but currently, it is unclear what contribution microglia and monocytes make. An apolipoprotein E mimetic peptide that is postulated to regulate microglial activation improves outcome after murine ICH and SAH and decreases microgliosis suggesting a role for microglia in the inflammatory cascade leading to damage after SAH [66]. Microglia surface area has been shown to be approximately 40% larger around vessels with microthrombosis than those without microthrombosis, which indicates that microthrombosis and microglia are interrelated and together contribute to the pathogenesis of DCI after SAH [65, 67].

Other cells in the brain parenchyma have the potential to initiate and regulate inflammation. Chief among them are astrocytes and OPCs. Both have been implicated in neuroinflammatory disorders such as multiple sclerosis [68, 69]. Evidence of astrocyte activation that correlates with cognitive deficits after SAH include cell surface changes in glial fibrillary protein (GFAP) and vimentin in animals with SAH that are reversed in MPO null mice that have an improved outcome [57]. Astrocyte proteins such as S-100b and GFAP are elevated in patients with SAH who develop DCI [70–73]. An autopsy study in humans found a higher intensity of GFAP staining and an increased GFAP surface coverage in the brain tissue of patients who died from SAH compared to the brain tissue of patients who died from other causes [16]. In addition, astrocytes regulate blood–brain barrier integrity, which is impaired in SAH [74, 75]. OPCs on the other hand, have barely been studied in the setting of SAH. Work on non-microglia glia is in its infancy; more studies are needed to determine what role astrocytes and OPCs play after SAH.

## Model of Inflammatory Changes in SAH

In the siloed approach to investigations of different inflammatory pathways in SAH, there has been an attempt to find out which cell or protein leads to poor outcome in the hopes of finding therapeutics. This overlooks the basic premise of the inflammatory system that it is a complex cascade of different actors, both cellular and proteinaceous, that interact over both space and time to coordinate the inflammatory response to SAH [76]. That this coordinated inflammatory response leads to the damaging downstream consequence of DCI likely suggests an evolutionary gap (people did not survive SAH 10,000 years ago, so there was no evolutionary pressure to regulate the inflammatory response) or a unique aspect of the central nervous system’s inflammatory response (what was previously referred to as immune privilege) that prevents inflammatory regulation to mitigate damage.

**Fig. 1:**
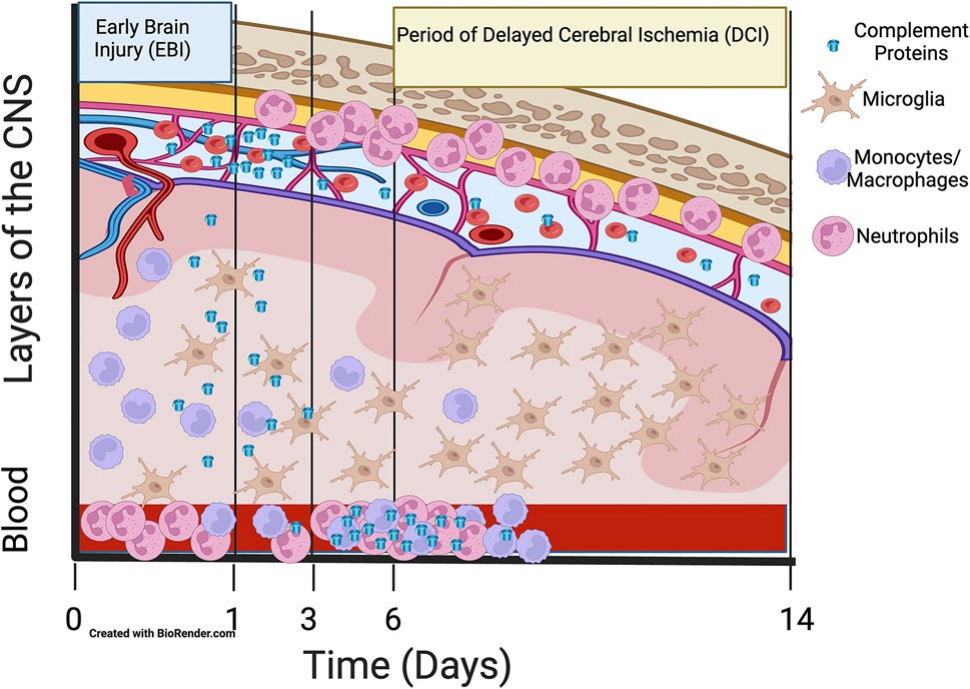
Time course of innate inflammatory cells and proteins after SAH extrapolated from preclinical and clinical data. There is early infiltration of the brain and CSF of monocytes and complement proteins. At 3 to 6 days in DCI, there is an accumulation of neutrophils in the meninges and the activation of microglia in the brain parenchyma. In the blood, neutrophils accumulate early and complement later in patients who develop DCI [37, 40, 42, 44, 46–48]

Using space and time to understand the response may lead to interesting testable questions around where in the inflammatory cascade therapeutics may play a role. From the data regarding the different inflammatory pathways, it is clear that inflammatory cell numbers in the blood differ from those in the CSF and brain [17, 59, 76]. We have constructed a space and time graphic that suggests aspects of the cascade and how the actors may interact (Fig. 1).

## Conclusion

Evidence suggests that the innate immune system plays an outsized role both early and in the days to weeks after SAH. Understanding the complex cascade that leads to damage may be critical to finding rational therapeutic targets to prevent DCI and improve outcome after SAH.

## Data Availability

No datasets were generated or analysed during the current study.
